# Multi‐slicing strategy for the three‐dimensional discontinuity layout optimization (3D DLO)

**DOI:** 10.1002/nag.2566

**Published:** 2016-08-17

**Authors:** Yiming Zhang

**Affiliations:** ^1^ Material‐Technology Innsbruck (MTI)University of InnsbruckTechnikerstraße 13Innsbruck6020Austria

**Keywords:** discontinuity layout optimization (DLO), modelling (pre‐processing), upper bound limit analysis, Mohr–Coulomb failure criteria

## Abstract

Discontinuity layout optimization (DLO) is a recently presented topology optimization method for determining the critical layout of discontinuities and the associated upper bound limit load for plane two‐dimensional and three‐dimensional (3D) problems. The modelling process (pre‐processing) for DLO includes defining the discontinuities inside a specified domain and building the target function and the global constraint matrix for the optimization solver, which has great influence on the the efficiency of the computation processes and the reliability of the final results. This paper focuses on efficient and reliable pre‐processing of the discontinuities within the 3D DLO and presents a multi‐slicing strategy, which naturally avoids the overlapping and crossing of different discontinuities. Furthermore, the formulation of the 3D discontinuity considering a shape of an arbitrary convex polygon is introduced, permitting the efficient assembly of the global constraint matrix. The proposed method eliminates unnecessary discontinuities in 3D DLO, making it possible to apply 3D DLO for solving large‐scale engineering problems such as those involving landslides. Numerical examples including a footing test, a 3D landslide and a punch indentation are considered, illustrating the effectiveness of the presented method. © 2016 The Authors. International *Journal for Numerical and Analytical Methods in Geomechanics* published by John Wiley & Sons Ltd.

## 1 Introduction

Topology optimization is a type of mathematical approach for searching for the optimum material layout considering a given space. In engineering fields, topology optimization is commonly used for obtaining the best design considering one or several given target variables such as weight/cost and a series of constraints such as specific loads and supports 1. With the progress of computing power in the last few decades, presently, it is possible to efficiently analyse large‐scale problems with millions of variables on normal personal computers 2, 3, 4.

Belonging to the family of topology optimization, discontinuity layout optimization (DLO) is a recently presented, elegant and promising numerical method for determining (i) the critical layout of discontinuities in a body at failure and (ii) the associated upper bound limit load 5 for plasticity problems 6, 7, 8, 9. Introducing large numbers of potential discontinuities permitted to crossover one another in a given domain and specifying the loading and boundary conditions (live loads, dead loads and supports), DLO can automatically generate a set of activated discontinuities corresponding to the upper bound limit load. This method has been successfully used to determine the slip‐line mechanisms of several geotechnical problems 7, 10, 11, the yield‐line failure of slabs 12, 13, failure mechanisms of masonry structures 14 and of retaining walls in seismic conditions 15.

In two‐dimensional (2D) DLO, the discontinuities are geometrically represented by line segments, simplifying the pre‐processing. Introducing large numbers of grid points into a specific domain, the potential discontinuities are built by connecting each pair of two grid points. In three‐dimensional (3D) DLO, on the other hand, the discontinuities are geometrically represented by polygons. Overlapping and crossing of different 3D discontinuities should be avoided to save computing resources 8, 9, as an important pre‐processing task. For the potential wide application of 3D DLO, a highly efficient pre‐processing method is necessary.

In this paper, the author presents a multi‐slicing strategy for the pre‐processing of discontinuities for 3D DLO, naturally avoiding the aforementioned overlap and crossing of different 3D discontinuities. Furthermore, a 3D discontinuity formulation with the shape of an arbitrary convex polygon is introduced, assuring the efficient assembly of the global constraint matrix. Computing effort during pre‐processing is saved while the number of unnecessary discontinuities is greatly reduced, paving the way for the analysis of large‐scale engineering problems such as landslides with 3D DLO.

This paper is organized as follows: in Section 2, DLO formulations for 2D and 3D problems, including the standard pre‐processing steps, will be presented, highlighting the necessity for a new method for pre‐processing of 3D DLO problems. The multi‐slicing strategy and the formulation of 3D discontinuity considering a shape of an arbitrary convex polygon will be presented in Section 3. In Section 4, a footing test, a 3D landslide problem and a punch indentation problem will be investigated, proving the effectiveness of the presented method. This paper closes with concluding remarks given in Section 5.

## 2 Basics of Discontinuity Layout Optimization

From this section on, the author denotes the local variable (vector, matrix) with a subscript, for example **d**_*i*_ represents the displacement jump of the discontinuity “*i*”. Meanwhile the symbol without subscript is used to represent a global variable (vector, matrix) such as **d** = [**d**_*i*_,⋯,**d**_*m*_]^*T*^ being the displacement jump of all the discontinuities in the domain. Furthermore, for better understanding, the dimensions of the equations are provided in the form of ℒ for length and F for force (mass·*length*·*time* ^−2^).

### 2.1 Kinematics

**Figure 1:**
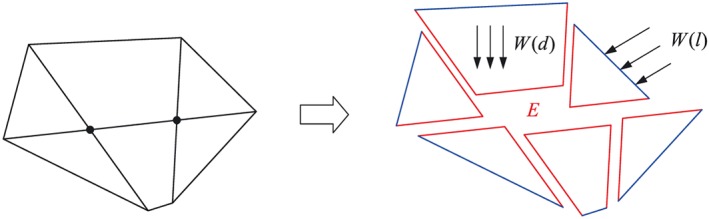
Potential discontinuities become activated at failure. [Colour figure can be viewed at wileyonlinelibrary.com]

The detailed formulation of the DLO was first presented in 7. For a domain with multiple potential discontinuities shown in Figure 1, several discontinuities become activated (i.e. displacement jump ≠ 0) during loading. When assuming rigid body displacements of the subdomains separated by the activated discontinuities, based on energy conservation, the following relationship is obtained:

(1) W(l)+W(d)−E=0,(F·ℒ)

where *W*(*l*) is the work performed by the live load, *W*(*d*) is the work performed by the dead load such as self weight and *E* is the energy dissipated at the discontinuities.

When minimizing the live load *W*(*l*) in the course of limit analysis, the target function of the optimization problem is obtained as

(2) $$W(l)=\left[E-W(d)\right]\to \min,\;(F\cdot ℒ)$$

**Figure 2:**
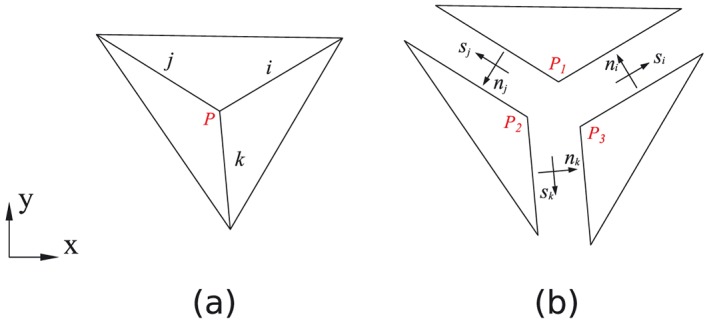
Two‐dimensional (2D) discontinuities connected to one node *P*: (a) before separation and (b) after separation. [Colour figure can be viewed at wileyonlinelibrary.com]

**Figure 3:**
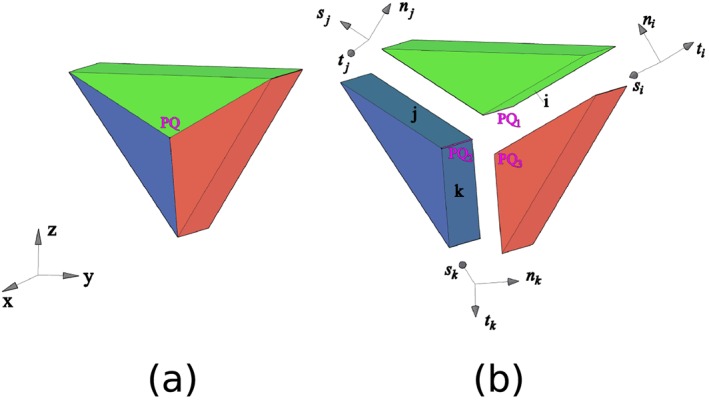
Three‐dimensional (3D) discontinuities connected to one edge *PQ*: (a) before separation and (b) after separation. [Colour figure can be viewed at wileyonlinelibrary.com]

In case several discontinuities are connected to one node, because of the rigid displacement of the subdomains, compatibility relationships are formulated at this node. For example, for the three 2D discontinuities *i*, *j* and *k* connected to the node *P*, with corresponding displacement jump ‡The author would like to emphasize that **d**_*i*_ should not be considered as ‘displacements of the discontinuity *i*’. **d**_*i*_ is the ‘displacement jump’ between the two subdomains separated by the discontinuity *i*. in the normal and shear directions §Rotational failure mechanisms are not considered in this paper, the formulation of which was given in 10.
**d**_*i*_=[*s*_*i*_,*n*_*i*_]^*T*^, **d**_*j*_=[*s*_*j*_,*n*_*j*_]^*T*^, and **d**_*k*_=[*s*_*k*_,*n*_*k*_]^*T*^(Figure 2), following relationship is obtained:

(3) $$X_{P}=\left[\begin{array}{ccc} B_{P,i} & B_{P,j} & B_{P,k} \end{array}\right]\left[\begin{array}{c} d_{i}\\ d_{j}\\ d_{k} \end{array}\right]=0,\;(ℒ)$$

where **X**_*P*_ is the relative displacement of node *P*, (**B**_*P*,*i*_ **d**_*i*_) is the contribution of discontinuity *i* to the relative displacement of node *P*. ¶For better understanding of Equation (3), the author suggests reverse process considering Figure 2(a)–(b). Because *P*_1_, *P*_2_ and *P*_3_ in Figure 2(b) come into the same position as *P* in Figure 2(a), the overall relative displacement of the node *P* contributed from the discontinuities *i*, *j* and *k* is equal to zero.
**B**_*P*,*i*_ is a coordinate transformation matrix (2 × 2 for 2D, see 7 for details and 3 × 3 for 3D, see Appendix A for details) transforming the local displacement jump **d**_*i*_ (given in local coordinates) to the global coordinate system 7, 8. In 3D DLO, this compatibility relationship is formulated for every edge 8, 9, such as edge *PQ* illustrated in Figure 3.

The compatibility relationships are built at every node (for 2D DLO) or edge (for 3D DLO) in the whole domain. A global compatibility constraint is obtained as

(4) $$X=\left[\begin{array}{c} X_{P}\\ \vdots \\ X_{L} \end{array}\right]=B\;d=\left[\begin{array}{ccc} B_{P,i} & \cdots & B_{P,m}\\ \vdots & \ddots & \vdots \\ B_{L,i} & \cdots & B_{L,m} \end{array}\right]\left[\begin{array}{c} d_{i}\\ \vdots \\ d_{m} \end{array}\right]=0,\;(ℒ)$$

where *P*,⋯,*L* are the nodes (for 2D DLO) or edges (for 3D DLO) of the domain, *i*,⋯,*m* are the discontinuities of the domain. **B** is a sparse matrix with row number 2 × *n* *p* (for 2D DLO, *np* is the total number of nodes) or 3 × *n* *e*(for 3D DLO, *ne* is the total number of edges) and column number 2 × *n* *d* (for 2D DLO) or 3 × *n* *d*(for 3D DLO), with *nd* being the total number of discontinuities.

### 2.2 Flow rule considering Mohr–Coulomb failure

**Figure 4:**
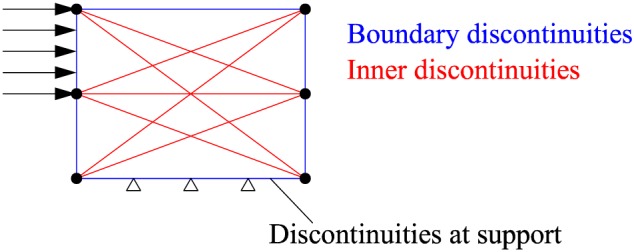
Two types of discontinuities considering geometrical positions: boundary and inner discontinuities. [Colour figure can be viewed at wileyonlinelibrary.com]

Considering the geometrical positions of the discontinuities, there are two types of discontinuities in the domain as (i) boundary discontinuity and (ii) inner discontinuity ∥It was pointed out in 7 that damage could happen at the supports as well. Therefore, 7 considered discontinuities at supports as inner discontinuities. In this paper, this type of damage is ignored for simplicity. Hence, the types of discontinuities depend only on their geometrical positions.(Figure 4).

Considering a discontinuity *j* with displacement jump as **d**_*j*_=[*s*_*j*_,*n*_*j*_]^*T*^ for 2D or **d**_*j*_=[*s*_*j*_,*t*_*j*_,*n*_*j*_]^*T*^ for 3D, if *j* is a boundary discontinuity, **d**_*j*_ equals to the absolute displacement. The activations of the boundary discontinuities do not contribute to the dissipated energy *E* (Figure 1).

If *j* is an inner discontinuity, on the other hand, **d**_*j*_≠0 indicates the sliding and opening of the subdomains separated by *j*. Considering an associated flow rule and Mohr–Coulomb failure, and the assumption of the rigid displacement of the subdomains, the following relationship is obtained as

(5) $$\begin{array}{cc} \text{2D:} & \;n_{j}-\tan\;\phi_{j}\cdot |s_{j}|=0\\ \text{3D:} & \;n_{j}-\tan\;\phi_{j}\cdot \sqrt{s_{j}^{2}+t_{j}^{2}}=0 \end{array},\;(ℒ)$$

where *ϕ*_*j*_ is the angle of friction (or dilation) of the inner discontinuity *j*. **Equation (5) indicates *n*_*j*_≥0, assuring the two subdomains separated by the discontinuity will not intrude into each other.


The authors of 7, 8 introduced a plastic multiplier **p**_*j*_=[*p*_*j*,1_,*p*_*j*,2_]^*T*^ (for 2D) or **p**_*j*_=[*p*_*j*_](for 3D) for transforming Equation (5) into the formulations respectively suitable for solution via linear programming (LP) and second‐order cone programming (SOCP) problems as follows:

(6) $$\begin{array}{c} \text{2D:}\left\{\begin{array}{c} N_{j}\left[\begin{array}{c} d_{j}\\ p_{j} \end{array}\right]=\left[\begin{array}{cccc} -1 & 0 & 1 & -1\\ 0 & -1 & \tan\;\phi_{j} & \tan\;\phi_{j} \end{array}\right]\left[\begin{array}{c} s_{j}\\ n_{j}\\ p_{j,1}\\ p_{j,2} \end{array}\right]=0\\ \text{with}p_{j,1}\geq 0,\;p_{j,2}\geq 0. \end{array}\right.\\ \text{3D:}\left\{\begin{array}{c} N_{j}\left[\begin{array}{c} d_{j}\\ p_{j} \end{array}\right]=\left[\begin{array}{cccc} 0 & 0 & -1 & \tan\;\phi_{j} \end{array}\right]\left[\begin{array}{c} s_{j}\\ t_{j}\\ n_{j}\\ p_{j} \end{array}\right]=0\\ \text{with}p_{j}\geq \sqrt{s_{j}^{2}+t_{j}^{2}}. \end{array}\right. \end{array}\;,\;\;\;(ℒ)$$

where $p_{j}\geq \sqrt{s_{j}^{2}+t_{j}^{2}}$ is the conic part in 3D DLO, and where the number of the cones equals the number of inner discontinuities.

Equation (6) indicates (*n*_*j*_=*p*_*j*_tan*ϕ*_*j*_) for 3D DLO, as also given in 8. By substituting *n*_*j*_ with (*p*_*j*_tan*ϕ*_*j*_) for every inner discontinuities *j*, great computing resources are saved (less unknowns regarding linear dependencies), bringing the formulation of 3D DLO later in Section 2.4.

For all inner discontinuities in the domain, the following constraint is obtained:

(7) $$N\left[\begin{array}{c} d\\ p \end{array}\right]=0,\;(ℒ)$$

### 2.3 Loading and boundary conditions

As mentioned in Section 2.1, the displacement field of the domain is described by **d**, with *W*(*d*) obtained as

(8) $$W(d)=f\;d=\left[\begin{array}{ccc} f_{i} & \cdots & f_{m} \end{array}\right]\left[\begin{array}{c} d_{i}\\ \vdots \\ d_{m} \end{array}\right],\;(F\cdot ℒ)$$

where **f**_*i*_ is the load vector (dead load) applied on discontinuity *i* 7.

For work performed by the live load *W*(*l*), the unit displacement constraint 7 is introduced as

(9) $$\begin{array}{cc} W(l) & =F\;(Ad),\;(F\cdot ℒ)\\ \text{with}\\ Ad & =\left[\begin{array}{ccc} A_{i} & \cdots & A_{m} \end{array}\right]\left[\begin{array}{c} d_{i}\\ \vdots \\ d_{m} \end{array}\right]=1,\;({ℒ}^{3}) \end{array}$$

where *F* is the unknown limit load $\left(F\cdot {ℒ}^{-2}\right)$, ††Conventionally, the dimensionless load factor *λ*(−) is optimized 7, 8, whereas in this paper the load ‘*F*’ is optimized, with the same value as *λ*, but with dimension $\left(F\cdot {ℒ}^{-2}\right)$, such that $F\;(F\cdot {ℒ}^{-2})=\lambda \;(-)\cdot 1\;(F\cdot {ℒ}^{-2})$. Furthermore, $W(l)\;(F\cdot ℒ)=F\;(F\cdot {ℒ}^{-2})\cdot 1\;({ℒ}^{3})$, based on Equation (9).
**A**_*i*_ is a vector taking into account the area of the discontinuity *i* (when *i* is loaded by the live load) and the projection of unit vector parallel to the live load on the discontinuity *i*.

Dissipated energy E is obtained by the plastic multiplier vector as

(10) $$E=ep=\left[\begin{array}{ccc} e_{j} & \cdots & e_{k} \end{array}\right]\left[\begin{array}{c} p_{j}\\ \vdots \\ p_{k} \end{array}\right],\;(F\cdot ℒ)$$

where **e**_*j*_ is the dissipated energy vector of the inner discontinuity *j*. Considering *c*_*j*_ as the cohesion of discontinuity *j*, **e**_*j*_=[*c*_*j*_ *l*_*j*_,*c*_*j*_ *l*_*j*_] (for 2D) with *l*_*j*_ being the length of discontinuity *j* or **e**_*j*_=[*c*_*j*_ *a*_*j*_](for 3D) with *a*_*j*_ being the area of the discontinuity *j*.

As mentioned in Section 2.2, for the boundary discontinuities, the displacement jump equals to the absolute displacement of the discontinuities, which is used for prescribing fixed supports, such as

- If the discontinuity *i* is fixed (supported) along the shear direction,set *s*_*i*_=0(for 2D) or *s*_*i*_=0,*t*_*i*_=0(for 3D);
- If the discontinuity *i* is fixed (supported) along the normal direction,set *n*_*i*_=0.

Another type of constraint commonly encountered is locked discontinuities. For example, if discontinuities *i* and *j* are locked together (**d**_*i*_=**d**_*j*_), the following constraint is added into the optimization formulation:

(11) $$L_{\mathrm{ij}}\left[\begin{array}{c} d_{i}\\ d_{j} \end{array}\right]=\left[\begin{array}{cc} I & -I \end{array}\right]\left[\begin{array}{c} d_{i}\\ d_{j} \end{array}\right]=0,\;(ℒ)$$

where **I** is the unit matrix.

### 2.4 Optimization formulation

Considering Equations (4), (7), (8), (9), (10) and (11), the optimization formulation (written in matrix form) for the 2D DLO problem is obtained as follows:

(12) $$\begin{array}{c} \text{minimize}\\ W(l)=E-W(d)=\left[\begin{array}{cc} -f & e \end{array}\right]\left[\begin{array}{c} d\\ p \end{array}\right],\;(F\cdot ℒ)\\ \text{subject to}\\ \left[\begin{array}{cc} B & 0\\ N\\ L & 0\\ A & 0 \end{array}\right]\left[\begin{array}{c} d\\ p \end{array}\right]=\left[\begin{array}{c} 0\\ 0\\ 0\\ 1 \end{array}\right],\;\left(\begin{array}{c} ℒ\\ ℒ\\ ℒ\\ {ℒ}^{3} \end{array}\right)\\ \text{with}\\ \to \text{If discontinuity}i\text{is fixed along the shear direction:}\\ \text{set}s_{i}=0\;;\\ \to \text{If discontinuity}i\text{is fixed along the normal direction:}\\ \text{set}n_{i}=0\;;\\ \to p\geq 0. \end{array}$$

As mentioned before, to save computing resources in 3D DLO, *n*_*j*_=*p*_*j*_tan*ϕ*_*j*_ is considered for every inner discontinuity *j*. After redefining **d**_*j*_=[*s*_*j*_,*t*_*j*_,*p*_*j*_]^*T*^ for every inner discontinuity *j*, the following optimization formulation (written in matrix form) for 3D DLO is obtained

(13) $$\begin{array}{c} \text{minimize}\\ W(l)=\left[\begin{array}{c} f^{\mathrm{new}} \end{array}\right]\left[\begin{array}{c} d^{\mathrm{new}} \end{array}\right],\;(F\cdot ℒ)\\ \text{subject to}\\ \left[\begin{array}{c} B^{\mathrm{new}}\\ L\\ A^{\mathrm{new}} \end{array}\right]\left[\begin{array}{c} d^{\mathrm{new}} \end{array}\right]=\left[\begin{array}{c} 0\\ 0\\ 1 \end{array}\right],\;\left(\begin{array}{c} ℒ\\ ℒ\\ {ℒ}^{3} \end{array}\right)\\ \text{with}\\ \to \text{If discontinuity}i\text{is a boundary discontinuity:}\\ d_{i}={\left[s_{i},t_{i},n_{i}\right]}^{T};\\ \to \text{If discontinuity}j\text{is an inner discontinuity:}\\ d_{j}={\left[s_{j},t_{j},p_{j}\right]}^{T}\text{,with}B_{j}^{\mathrm{new}}=B_{j}\left[\begin{array}{c} I\\ \tan\;\phi_{j} \end{array}\right]\\ \text{and}p_{j}\geq \sqrt{s_{j}^{2}+t_{j}^{2}};\\ \to \text{If discontinuity}i\text{is fixed along the shear direction:}\\ \text{set}s_{i}=0\text{and}t_{i}=0;\\ \to \text{If discontinuity}i\text{is fixed along the normal direction:}\\ \text{set}n_{i}=0. \end{array}$$

where **f** ^*new*^ and **A** ^*new*^ are transformed from [−**f** **e**] and **A** by considering the redefinition of **d**_*j*_=[*s*_*j*_,*t*_*j*_,*p*_*j*_]^*T*^ and *n*_*j*_=*p*_*j*_tan*ϕ*_*j*_ for every inner discontinuity *j*. Equation (13) is equivalent to the original formulation given in 8.

Equations (12) and (13) can be solved respectively using LP and SOCP solvers. As recommended by 7, 8, the author also uses the software ‘Mosek’ for solving the optimization problems, see 16 for details.

### 2.5 Standard pre‐processing in discontinuity layout optimization

**Figure 5:**
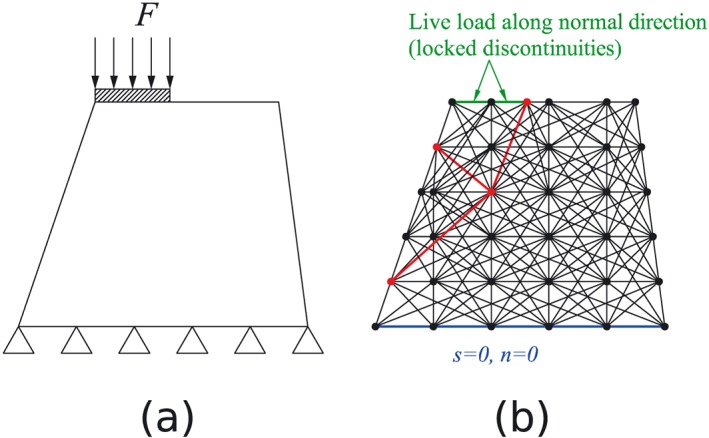
Fully connected grid method for the two‐dimensional (2D) discontinuity layout optimization (DLO) (a landslide example): (a) model for analysis and (b) fully connected model for optimization (only a subset of the discontinuities is shown for sake of clarity). [Colour figure can be viewed at wileyonlinelibrary.com]

A fully connected grid method is used for pre‐processing of 2D DLO problems 7. First, grid points are given in the 2D domain as nodes, with every two nodes connected as a discontinuity. The loading and boundary conditions will be set at the corresponding discontinuities. The activated discontinuities giving minimum limit load *F* will be automatically obtained after optimization, as illustrated in Figure 5.

**Figure 6:**
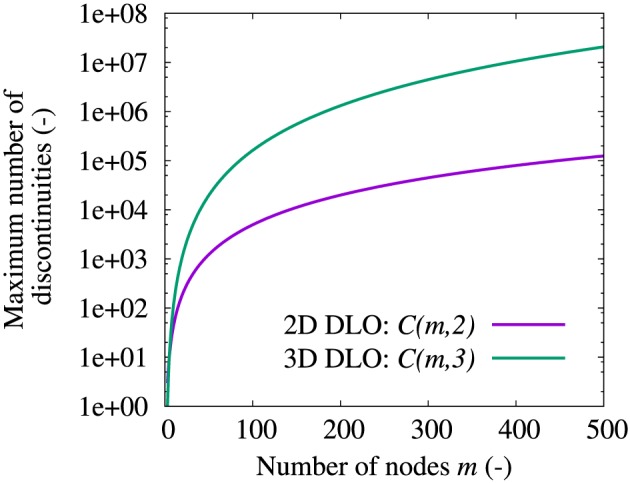
Maximum number of two‐dimensional (2D) and three‐dimensional (3D) discontinuities as functions of the number of nodes *m* when using fully connected grid method. [Colour figure can be viewed at wileyonlinelibrary.com]

This method is very direct and elegant in 2D DLO, with the maximum number of discontinuities equal to *C*(*m*,2) (*m* is the number of nodes) considering the mathematical combinations available. On the other hand, when using a similar method for 3D DLO, that is, considering nodes in a 3D domain and connecting every three nodes as a 3D discontinuity, the maximum number of discontinuities becomes *C*(*m*,3), which dramatically increases with increasing *m* (Figure 6).

### 2.6 The overlap and crossing of discontinuities in discontinuity layout optimization

**Figure 7:**
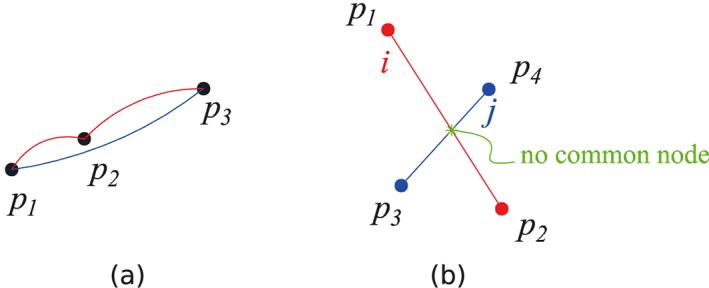
Overlapping and crossing of two discontinuities in two‐dimensional (2D) discontinuity layout optimization (DLO): (a) overlapping of two discontinuities when three nodes are on the same line (discontinuities shown as curves for sake of clarity) and (b) crossing of two 2D discontinuities (no common node located at the intersection point). [Colour figure can be viewed at wileyonlinelibrary.com]

**Figure 8:**
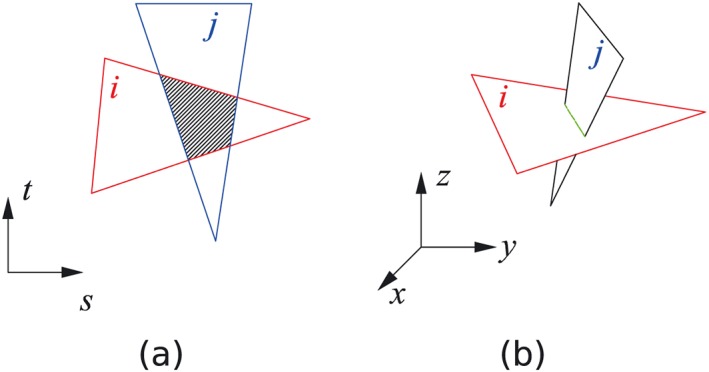
Overlapping and crossing of two discontinuities in three‐dimensional (3D) discontinuity layout optimization (DLO): (a) overlapping of two discontinuities when these two discontinuities are on the same plane and (b) crossing of two discontinuities on different planes. [Colour figure can be viewed at wileyonlinelibrary.com]

In the DLO, the overlap and crossing of the different discontinuities are commonly encountered, as illustrated in Figures 7 and  8.

**Figure 9:**
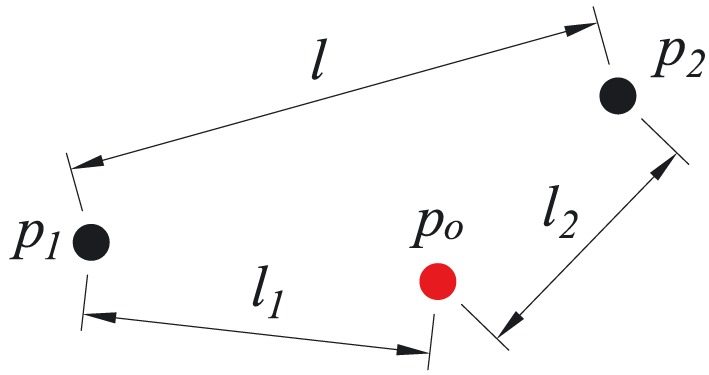
Distance checking to avoid overlapping of discontinuities in two‐dimensional (2D) discontinuity layout optimization (DLO). [Colour figure can be viewed at wileyonlinelibrary.com]

Overlapping of discontinuities may give incorrect displacement jumps, ‡‡The author uses the word “may” because the overlap of the discontinuities will not affect the final solution if these discontinuities are not activated. because displacement jumps are ‘shared’ in overlapped discontinuities. Moreover, overlapping of discontinuities will introduce large numbers of unnecessary discontinuities into the domain. In 2D DLO, distance checking is used to avoid the overlap of discontinuities. Two nodes *p*_1_ and *p*_2_ will only be connected by a discontinuity if |*l* − *l*_1_−*l*_2_|>*ε*(*ε* is a prescribed small value, and *p*_*o*_ is all the other nodes except *p*_1_ and *p*_2_) for assuring no other nodes are located between *p*_1_ and *p*_2_(Figure 9).

**Figure 10:**
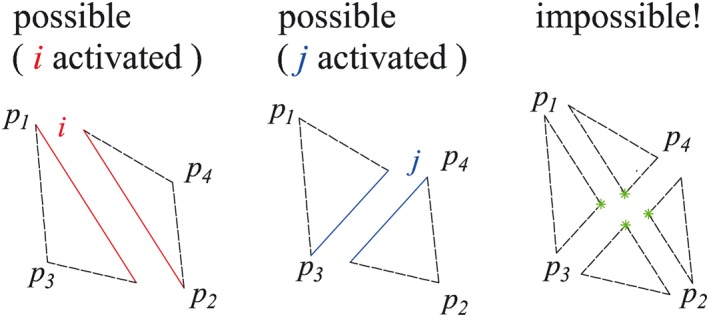
Possible and impossible deformation patterns of the subdomains separated by two crossed two‐dimensional (2D) discontinuities *i* and *j* shown in Figure 7(b). [Colour figure can be viewed at wileyonlinelibrary.com]

The presence of discontinuities, which cross over, does not affect the numerical stability of DLO. The fact that discontinuities can cross over one another is a great advantage of this method over other limit analysis methods (e.g. the finite element method 17, when using rigid elements) because of the resulting very wide search space. What should be borne in mind is that crossed discontinuities are not directly connected. Because there is no common node located at the intersection point, the geometrical compatibility relationship (Equation (3)) will not be built for the crossed discontinuities. Therefore, only limited failure patterns are available for crossed discontinuities (Figure 10). Hence, in 2D DLO, to take into account large numbers of failure patterns, it is necessary to use a fine grid and ensure there are enough nodes and corresponding fully connected discontinuities 7.

Considering the 3D situation, obviously distance checking cannot be used to avoid the overlap of two 3D discontinuities. In addition, introducing more nodes into the 3D domain and building a set of fully connected 3D discontinuities will cause a huge increase in the number of discontinuities (Figure 6). The only possible routine is to use limited nodes and properly build 3D discontinuities, which are not overlapped or crossed, thereby avoiding unnecessary discontinuities as well as taking into account a large number of possible failure patterns.

**Figure 11:**
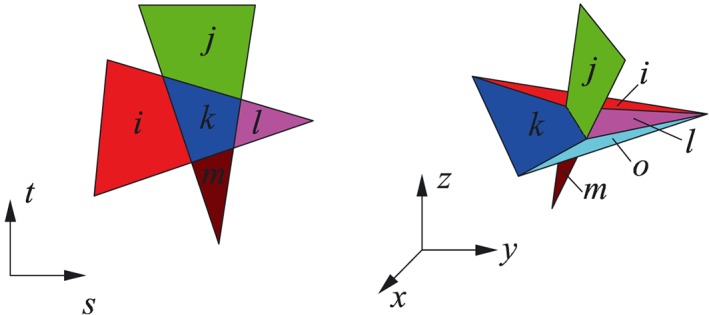
Adding nodes and splitting overlapped and crossed three‐dimensional (3D) discontinuities into more discontinuities not overlapped or crossed. [Colour figure can be viewed at wileyonlinelibrary.com]

**Figure 12:**
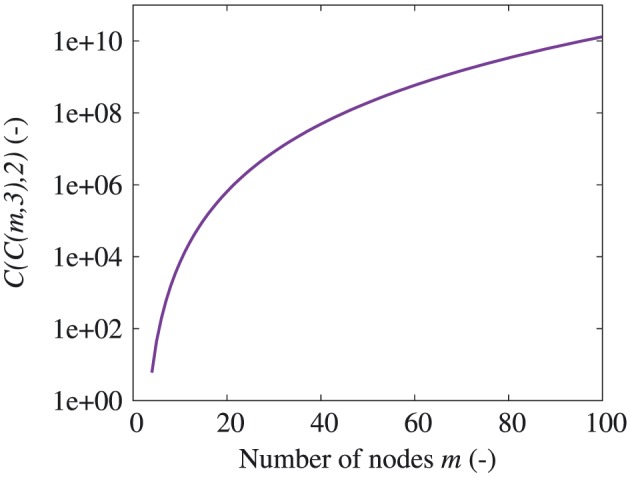
The number of times pairwise checking is required (one round) *C*(*C*(*m*,3),2) as a function of the number of nodes *m*. [Colour figure can be viewed at wileyonlinelibrary.com]

Presently, the standard method for dealing with the overlap and crossing of 3D discontinuities is to check every two 3D discontinuities in the domain then add more nodes and split the relevant discontinuities into more discontinuities 8, 9 (Figure 11). With such type of pairwise checking, the number of times of this checking (one round) is about *C*(*C*(*m*,3),2), with *m* being the number of nodes. This process would occupy great computing resources to conduct on a personal computer when the number of the nodes exceeds 100 (Figure 12). Furthermore, because new discontinuities are introduced during every round of the checking, several rounds of this checking process are necessary within every round, and more discontinuities need to be checked than in the last round. The standard pre‐processing of 3D DLO greatly restrains the potential wide application of this promising method. §§Wisely, in the numerical examples shown in 8, the domain was first discretized with 3D tetrahedron elements (similar to a discretization used in finite element method), then the surfaces of the 3D elements were taken as discontinuities (the overlap and crossing of discontinuities are avoided) for 3D DLO. Such strategy is suitable for some special cases, and the reliability of the results depend on the discretization of the domain, see the example given in Section 4.3. A more efficient strategy for modelling 3D discontinuities is essential for 3D DLO.

## 3 Multi‐Slicing Strategy

### 3.1 Geometrical relationship between slicings and three‐dimensional discontinuities

**Figure 13:**
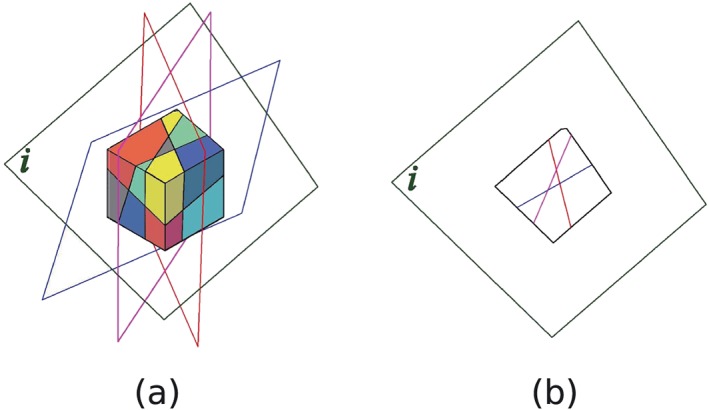
A domain cut by multiple slicings (outside surfaces of the domain are also considered as slicings): (a) the domain and the multiple slicings and (b) the section of the domain considering slicing *i*. [Colour figure can be viewed at wileyonlinelibrary.com]

A slicing is a plane, cutting the whole domain into two subdomains. Assuming the outside surfaces of a domain are also slicings and considering a domain cut by multiple slicings into several subdomains, the section of the domain of a specific slicing is composed of several convex polygons (Figure 13).

**Figure 14:**
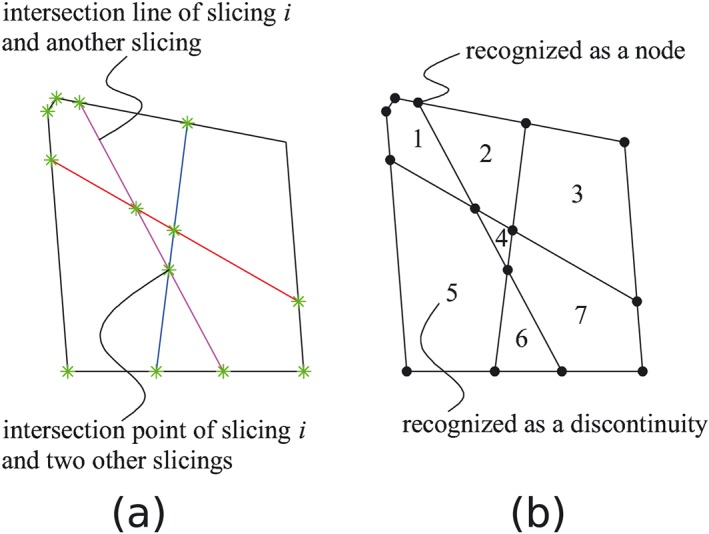
Section of domain considering a slicing *i*: (a) points and lines lying on slicing *i* and (b) points and convex polygons formed by intersected lines, which later are recognized as nodes and discontinuities (14 nodes and seven discontinuities recognized in this figure). [Colour figure can be viewed at wileyonlinelibrary.com]

Inspecting the section of a domain considering a slicing *i* as shown in Figure 14, every line is an intersection line of the slicing *i* and another slicing and every point is an intersection point of the slicing *i* and two other slicings. If the convex polygons formed by these lines and points are recognized as discontinuities, overlapping and crossing of the discontinuities are automatically avoided. Moreover, because every edge of the discontinuities is located on the intersection lines of the slicings, the displacement jump of the discontinuities on the corresponding slicings can be transferred to each other considering the geometrical compatibility relationship.

**Figure 15:**
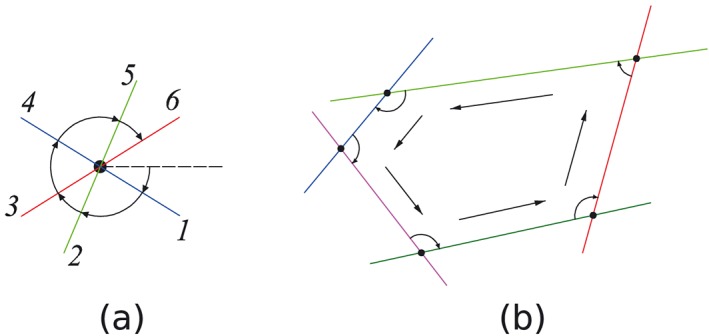
Recognition of convex polygons from several intersected lines: (a) ordering the intersected half lines with polar angle (clockwise) at every node and (b) search routine for recognizing a convex polygon formed by multiple intersected lines. [Colour figure can be viewed at wileyonlinelibrary.com]

Considering recognizing the convex polygons on one plane from several intersected lines, some highly efficient algorithms are available such as in 18. In this paper, on the other hand, the author uses a simple algorithm for this problem as illustrated in Figure 15. First, at every node, the intersected half lines are ordered with the polar angle (clockwise suggested by the author, bringing convenience for further building 3D discontinuities). Then, a start node and a search direction are specified, with the search direction turning to the neighbouring polar angle when coming to the next node. Once the search node comes to the start node again, a polygon is recognized. This process is repeated until every polygon has been recognized.

### 3.2 Three‐dimensional discontinuity considering shape of an arbitrary convex polygon

**Figure 16:**
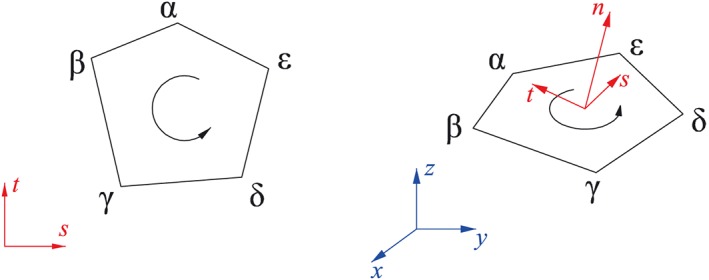
A three‐dimensional (3D) discontinuity ‘*o*’ formed by a node loop. [Colour figure can be viewed at wileyonlinelibrary.com]

When a convex polygon is recognized, a corresponding 3D discontinuity is defined by a node loop such as (*α*,*β*,⋯,*ϵ*) (where, *α*,*β*,⋯,*ϵ* are the id of the nodes), with the direction of the normal displacement jump *n* determined by the right hand rule (Figure 16). For 3D discontinuities on the same slicing, the author suggests a unified local coordinate system of *n*, *s* and *t* because of the associated easy coding during the programming processes (Appendix A). In addition, for discontinuities on the surfaces (boundary discontinuities), it is very important to assure the direction of *n* is defined from the outside of the domain point to the inside of the domain.

Considering geometrical compatibility in 3D DLO, there are two directions for each edge (such as in Figure 8, directions *P*→*Q* and *Q*→*P*)^8^. Hence, the sign should be determined during the assembly of the compatibility matrix **B**(Section 2.1). In this paper, the sign is determined by the id of the nodes in the node loop. For example, considering discontinuity ‘*o*’ shown in Figure 16, the local coordinate transformation matrix is **B**_*o*_. The contribution of discontinuity *o* to the relative displacement of the edge *α* − *β* is **X**_*α* − *β*,*o*_=**B**_*o*_ **d**_*o*_. When assembling **B**_*o*_ into **B**(Equation (4)), the sign is determined by the id of the two nodes of the edge as

(14) $$\begin{array}{cc} \;\;\text{column for}d_{o}\\ \;\;\;↓\\ B & =\left[\begin{array}{ccc} \ddots & \cdots & \ddots \\ \vdots & s\cdot B_{o} & \vdots \\ \ddots & \cdots & \ddots \end{array}\right]\leftarrow \text{row for}X_{\alpha -\beta }\\ \text{with}\;s & =\left\{\begin{array}{cc} 1 & \text{if}\alpha <\beta \\ -1 & \text{if}\alpha >\beta \end{array}\right. \end{array}$$

The multi‐slicing procedure for 3D DLO is summarized as follows:

1. Multiple slicings are prescribed;
2. Based on the multiple slicings, intersection points of every three non‐parallel slicings are calculated and defined as nodes;
3. All nodes on one specific slicing are collected; then all convex polygons on this slicing formed by intersection lines are recognized as 3D discontinuities. This process is repeated until all slicings are checked;
4. With the recognized 3D discontinuities and the constraint given in Equation (14) assembled, the optimization formulation (SOCP problem, see Equation (13)) is built and sent to the optimization solver for solving.

## 4 Numerical examples

A personal computer with Intel Core i7‐3930K 3.20G Hz processor and 16 GB memory was used to obtain the numerical results. The SOCP problem (Equation (13)) is built via the multi‐slicing strategy presented then solved by Mosek 7.1 16.

### 4.1 Footing problem

**Figure 17:**
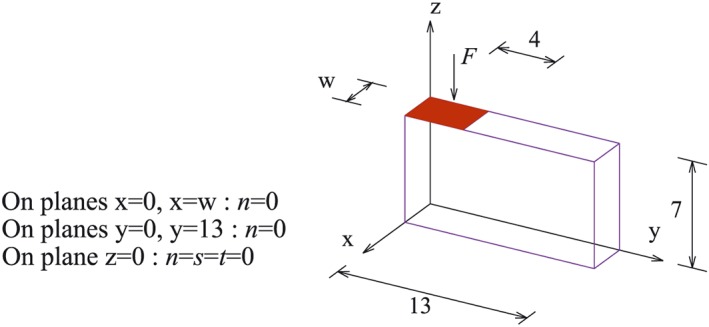
Model of the footing problem. [Colour figure can be viewed at wileyonlinelibrary.com]

The weightless footing problem 19 is shown in Figure 17, with the live load ‘*F*’ applied perpendicular on the marked region. When assuming the angle of friction *ϕ* = 0° and unified cohesion *c* for all discontinuities, the analytical solution for the limit loading ‘*F*’ is equal to (2 + *π*)*c*.

**Figure 18:**
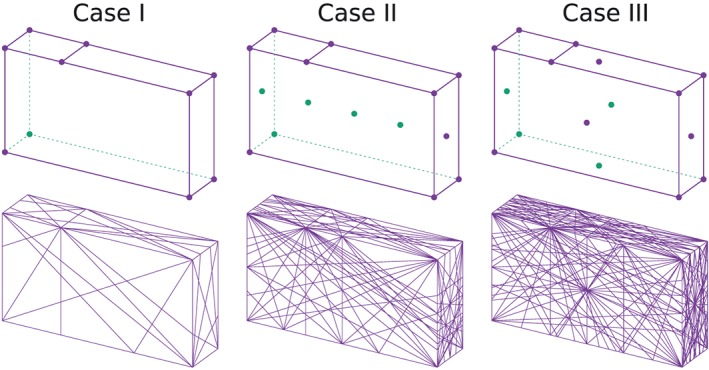
The discontinuities in the footing problem: key points prescribed for slicings (every three non‐collinear key points providing one slicing) and discontinuities built with multi‐slicing method (only visible discontinuities are shown). [Colour figure can be viewed at wileyonlinelibrary.com]

**Figure 19:**
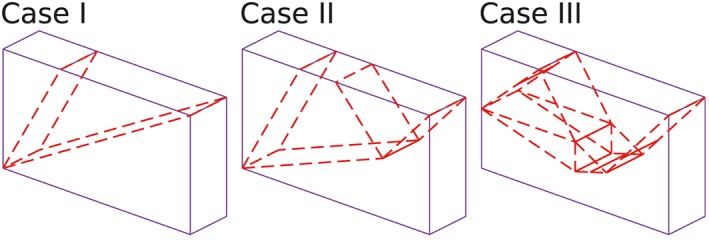
Activated discontinuities for the footing problem. [Colour figure can be viewed at wileyonlinelibrary.com]

Three cases with different slicings are considered (Figure 18). Key points are the points prescribed for defining the slicings, with every three non‐collinear points providing a slicing. ¶¶The footing problem shown in this paper is actually a 2D problem and only the discontinuities perpendicular to *y*–*z* plane will finally become activated. However, during the calculation, the author considers a general condition with all discontinuities given by the slicings taken into account. The details of the calculations are given in Table 1. ∥∥The outside surfaces of the domain are also considered as slicings (six slicings in total) in Table 1. Activated discontinuities are shown in Figure 19. The results indicate the applicability of the presented method. With the increasing number of slicings, the results are improved comparing to the analytical solution, with acceptable time cost for modelling. Theoretically, when introducing very large number of slicings, very accurate results would be obtained.

**Table 1:** Details of calculations for the footing problem.

	Case I	Case II	Case III
Number of slicings	40	93	137
Number of nodes	847	21837	66 554
Number of discontinuities	3652	78612	243080
Number of edges	3101	72276	221665
CPU time for modelling	0.11 s	17.24 s	155.46 s
(pre‐processing)			
CPU time for solving SOCP	3.93 s	1081.69 s	11535.81 s
Number of slicings with	8	10	14
activated discontinuities			
*F*	6.81*c*	6.16*c*	5.92*c*
Error	32.4%	19.8%	15.1%

SOCP, second‐order cone programming.

Nevertheless, unlike the LP problem used in 2D DLO, the SOCP problem is much more difficult to solve, taking a long time (Table 1) and occupying great computing resources (e.g. around 10 GB memory are occupied by Mosek when solving case III), which increases dramatically with increasing number of slicings. Besides, introducing more slicings will reduce the distance between nodes. When some nodes are ‘too close’ to each other considering the computational precision of the variables stored on the computer system, linear dependencies will be introduced in the global constraint matrix, greatly affecting the numerical stability of the optimization solver. ***Sweeping and combing nodes too close to each other could avoid the linear dependency and numerical stability problems, while on the other hand possibly resulting in slightly zigzagged slicings (some nodes of the slicing moved away from the original slicing plane) and unreliable results.


Considering the computational cost and assuring the numerical stability of the solver, presently, a number of slicings less than 100 is suitable, which is unfortunately not enough to obtain a mighty accurate result. For better results, some techniques considering engineering properties of the case should be used, for example, taking advantage of symmetry and introducing more slicings in the region close to the live load.

### 4.2 Three‐dimensional landslide

**Figure 20:**
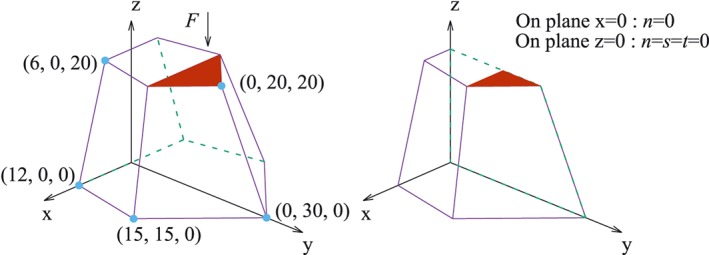
Model of the three‐dimensional (3D) landslide. [Colour figure can be viewed at wileyonlinelibrary.com]

**Figure 21:**
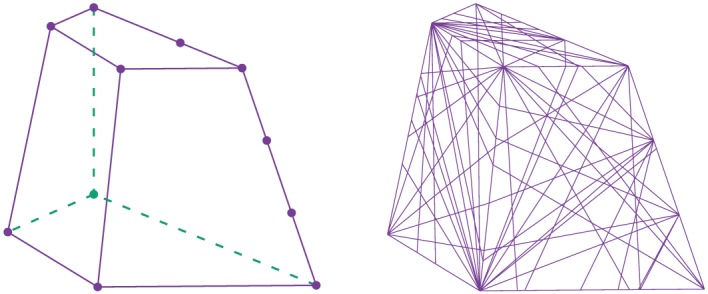
The discontinuities of the the three‐dimensional (3D) landslide: key points prescribed for slicings (every three non‐collinear key points providing one slicing) and discontinuities built with multi‐slicing method (only visible discontinuities are shown). [Colour figure can be viewed at wileyonlinelibrary.com]

**Figure 22:**
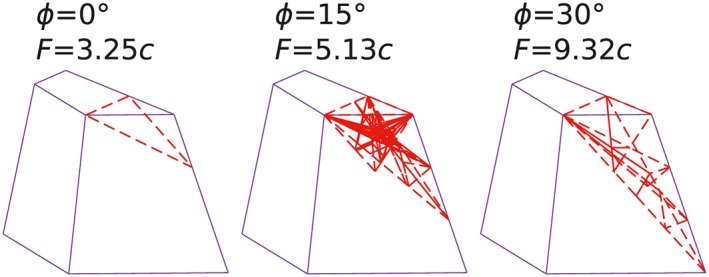
Activated discontinuities and associated limit loads *F* for the three‐dimensional (3D) landslide with the discontinuities shown in Figure 21. [Colour figure can be viewed at wileyonlinelibrary.com]

A weightless 3D landslide example is shown in Figure 20, with uniform cohesion *c* assumed. Owing to symmetry, only half of the problem is considered. The prescribed key points and the corresponding discontinuities are shown in Figure 21. Considering angle of frictions *ϕ* = 0°, *ϕ* = 15° and *ϕ* = 30°, the corresponding activated discontinuities and limit loads of the 3D landslide problem are shown in Figure 22, indicating a large failure zone and the possibility that the limit loads are overestimated.

**Figure 23:**
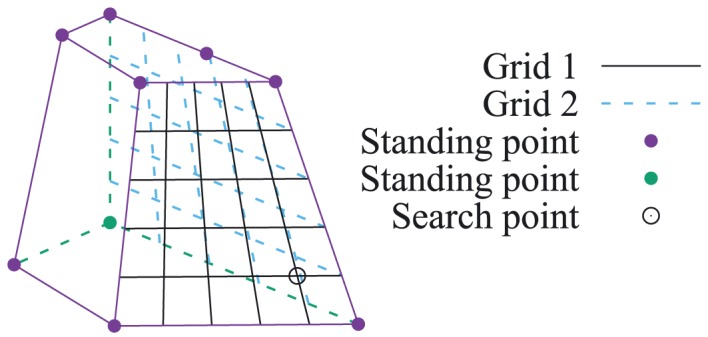
Grids used for the search procedure for the three‐dimensional (3D) landslide (only 5 × 5 grids are shown for the sake of clarity when actually 50 × 50 grids are used in the numerical analysis). [Colour figure can be viewed at wileyonlinelibrary.com]

Considering the failure pattern of the 3D landslide that many activated discontinuities appear at the loaded region, the author implemented a simple grid searching procedure into the multi‐slicing strategy to obtain better results. Several standing points are prescribed, then a search point is added, which moves on the grid (Figure 23). The multiple slicings include the surfaces and the slicings defined by the search point and another two standing points (three points are non‐collinear). In one search round, after all points on the grid are searched and calculated, the search point with minimum energy/limit load will be added as a new standing point; meanwhile, the corresponding slicings with activated discontinuities are also kept in the next search round.

**Figure 24:**
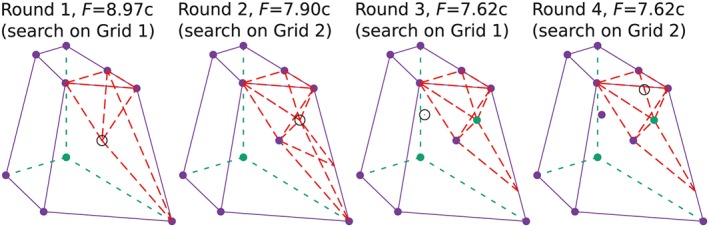
Considering *ϕ* = 30°, showing the activated discontinuities, the standing points and search point at the end of each search round with the grid searching procedure. [Colour figure can be viewed at wileyonlinelibrary.com]

**Figure 25:**
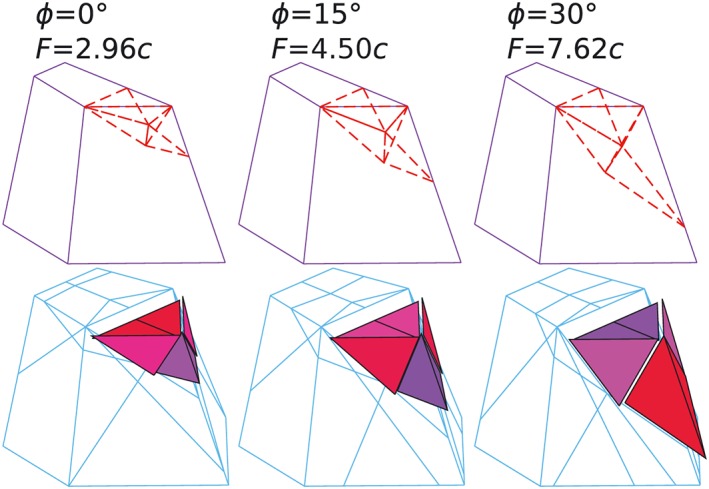
The obtained activated discontinuities, deformation patterns and associated limit loads *F* for the three‐dimensional (3D) landslide problem with the implemented grid searching procedure. [Colour figure can be viewed at wileyonlinelibrary.com]

With two 50 × 50 grids (Figure 23) and two search rounds on each grid, †††Two grids are searched, first grid 1 then grid 2. This process was run twice (two search rounds for each grid). considering *ϕ* = 30°, the obtained activated discontinuities, the standing points and search point in the end of every search round are shown in Figure 24, indicating the results are greatly improved with this search procedure. The final obtained minimum limit loads and failure patterns for the 3D landslide considering *ϕ* = 0°, *ϕ* = 15° and *ϕ* = 30° are shown in Figure 25, which are more reasonable than the results given in Figure 22.

### 4.3 Punch indentation

**Figure 26:**
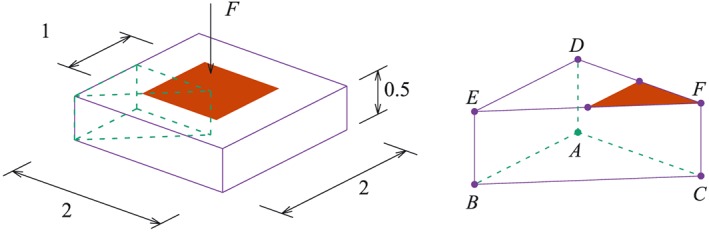
Model of the punch indentation. [Colour figure can be viewed at wileyonlinelibrary.com]

As a classic example, the punch indentation analysed in 8, 20, 21 is reanalysed here. A block with its bottom fixed and lateral surfaces restrained along the normal direction (*n* = 0) is loaded in the centre. Only 1/8 of the volume is considered, owing to symmetry (Figure 26), with uniform cohesion *c* considered.

**Table 2:** Punch indentation: comparisons with benchmark solutions (upper bound).

			CPU		CPU
Provided by	Methods	*ϕ* = 0°	time	*ϕ* = 30°	time
20	Multi‐block analysis	*F* = 6.561*c*	—	*F* = 104.019*c*	—
21	FEM limit analysis	*F* = 6.051*c*	—	*F* = 66.936*c*	—
8	3D DLO	*F* = 6.226*c*	6400 s	—	—
This work	3D DLO (cube – 1/4)	*F* = 6.433*c*	0.1 s	—	—
This work	3D DLO (cube – 1/16)	*F* = 6.419*c*	140 s	—	—
This work	3D DLO (cube – 1/24)	*F* = 6.418*c*	4371 s	—	—
This work	3D DLO (5× multi‐slicing)	*F* = 6.094*c*	2037 s	*F* = 73.196*c*	6249 s
This work	3D DLO (10× multi‐slicing)	*F* = 5.904*c*	11144 s	*F* = 69.520*c*	38035 s
This work	3D DLO (25× multi‐slicing)	*F* = 5.892*c*	80322 s	*F* = 68.225*c*	272156 s

**Figure 27:**
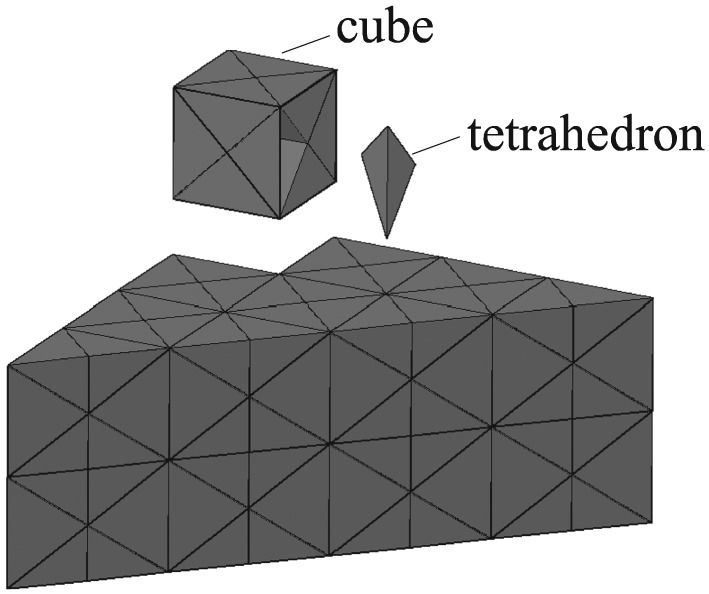
Discretization of the domain with a regular pattern of cubes subdivided into 24 tetrahedra (cube – 1/4 is shown). [Colour figure can be viewed at wileyonlinelibrary.com]

As mentioned before, overlapping and crossing problems can also be avoided if discontinuities are transformed from the surfaces of 3D elements of a discretized domain. For testing this strategy, the model is discretized with tetrahedra (similar discretization is used in finite element method analysis in 22) and the surfaces of the tetrahedra are transformed into discontinuities (Figure 27). The results are listed in Table 2, ‡‡‡Because the CPU time listed in Table 2 referring the work of 8 was obtained with a different computer, direct comparison could be unfair. regarding the edge length of the cube equals 1/4(denoted as cube – 1/4), 1/16 (denoted as cube – 1/16) and 1/24 (denoted as cube – 1/24). From the results, it can be found that one drawback of this strategy is that a finer mesh does not ensure a corresponding much better result, but does take much more computing time.

**Figure 28:**
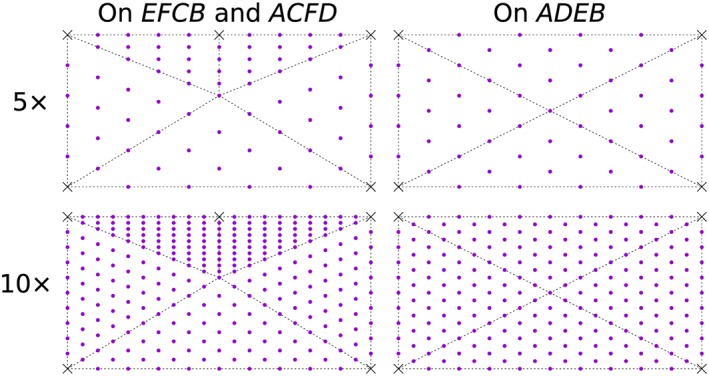
Grid points for grid searching procedure (only 5× and 10× are shown). [Colour figure can be viewed at wileyonlinelibrary.com]

**Figure 29:**
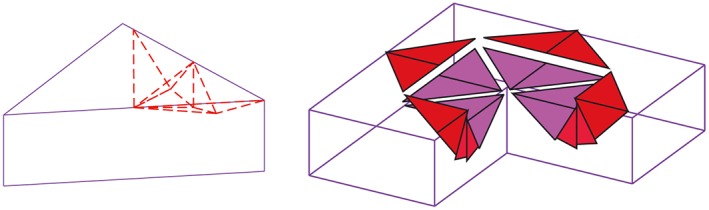
Obtained activated discontinuities and deformation pattern for the punch indentation problem regarding *ϕ* = 0° and 25× grid. [Colour figure can be viewed at wileyonlinelibrary.com]

Then the multi‐slicing method with grid searching procedure is used. The initial standing points for the search procedure are shown in Figure 26. In this example, grids similar to cobweb are prescribed on the surfaces *EFCB*, *ACFD* and *ADEB* (Figure 28), with four search rounds used (each surface is searched four times). Obtained and literature results are listed in Table 2, with the obtained activated discontinuities and deformation pattern regarding *ϕ* = 0° and 25× grid shown in Figure 29. It can be seen that using the multi‐slicing method combing with the search procedure provides satisfying results even with 5× grid, which takes much less computing time than the case cube – 1/24. Furthermore, the results are continuously improved with finer search grid, indicating the effectiveness and robustness of this method.

## 5 Conclusion

In this paper, in order to avoid overlapping and crossing of 3D discontinuities, a multi‐slicing strategy was presented, with 3D discontinuities being arbitrary convex polygons. The presented method has been proven to be effective, and numerical results are improved with increasing numbers of slicings. Although the number of slicings is still limited because of the complexity at solving large scale SOCP problems, considering the engineering properties of the cases, the author took the advantage of the symmetry and implemented a grid searching procedure, obtaining much better results. The work proposed in this paper opens the door to the application of the 3D DLO to some large scale engineering problems.

## Acknowledgements

The author gratefully acknowledges the financial support provided by the Austrian Science Fund (FWF), grant no. P 25614 ‘Adaptive Discontinuity Layout Optimization’.

## Figures and Tables

**Figure A.1:**
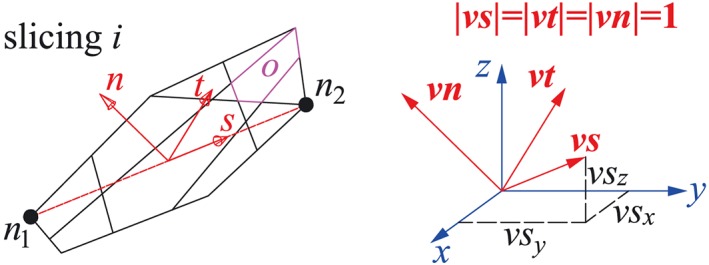
Three‐dimensional (3D) discontinuity *o* lying on the corresponding slicing *i*: local and global coordinate system. [Colour figure can be viewed at wileyonlinelibrary.com]

## Appendix A The Coordinate Transformation Matrix in Three‐Dimensional Discontinuity Layout Optimization

### A.1

As mentioned in Section 3, the author suggests a unified local coordinate system for discontinuities on the same slicing, which makes the compatibility matrix for all the discontinuities lying on this slicing the same, bringing great convenience for coding. Figure A.1 illustrates a 3D discontinuity *o* lying on slicing *i* with the corresponding local coordinate system *s*,*t*,*n* and global coordinate system *x*,*y*,*z*. When *n* being perpendicular to the slicing *i*, *s* is determined by two farthest nodes *n*_1_ and *n*_2_ lying on the slicing *i*.

Assuming the unit vectors parallel to *s*,*t*,*n* are defined as *v* *s*,*v* *t*,*v* *n*, the coordinates of *v* *s*,*v* *t*,*v* *n* in *x*,*y*,*z* system are (*v* *s*_*x*_,*v* *s*_*y*_,*v* *s*_*z*_), (*v* *t*_*x*_,*v* *t*_*y*_,*v* *t*_*z*_) and (*v* *n*_*x*_,*v* *n*_*y*_,*v* *n*_*z*_). Considering a change of basis, the compatibility matrix of *o* as **B**_*o*_ (Equation (14)) is determined by

(15) $$B_{o}={\left[\begin{array}{lll} vs_{x} & vs_{y} & vs_{z}\\ vt_{x} & vt_{y} & vt_{z}\\ vn_{x} & vn_{y} & vn_{z} \end{array}\right]}^{-1},\;(-)$$
